# Long-term survival in a patient with non-small cell lung cancer harboring *KRAS* G13C and *TP53* co-mutations: case report and literature review

**DOI:** 10.3389/fmed.2026.1784222

**Published:** 2026-03-16

**Authors:** Ran Chen, Ziqi Ma, Mengyan Yuan, Kang Qian, Hongbin Zhu

**Affiliations:** Department of Respiratory and Critical Care Medicine, The Fourth Affiliated Hospital of Anhui Medical University, Chaohu, Anhui, China

**Keywords:** anti-angiogenic therapy, immune-combined therapy, *KRAS* G13C, lung adenocarcinoma, platinum-based doublet chemotherapy

## Abstract

*KRAS* mutations are frequent oncogenic drivers in non-small cell lung cancer (NSCLC). Although targeted therapies have revolutionized treatment for the G12C subtype, the G13C variant lacks approved specific agents and correlates with a poor prognosis. We report a 59-year-old male with locally advanced (stage IIIA) lung adenocarcinoma harboring concurrent *KRAS* G13C and *TP53* mutations. Surgery was contraindicated due to poor pulmonary function. The patient received first-line and maintenance therapy comprising carboplatin/pemetrexed, camrelizumab, and Endostar/bevacizumab. This regimen was well-tolerated and yielded a progression-free survival (PFS) exceeding 55 months. Of note, following regional lymph node progression, re-challenge with the original combination restored disease stability. Our findings suggest that the combination of chemotherapy, immunotherapy, and anti-angiogenic agents may represent a viable therapeutic strategy for patients with *KRAS* G13C/*TP53* co-mutated NSCLC. This case report suggests a potentially promising therapeutic strategy to improve long-term survival in this difficult-to-treat patient population.

## 1 Introduction

Lung cancer ranks first in global cancer incidence and mortality (1). The non-small cell subtype (NSCLC) accounts for approximately 85% of all diagnoses, representing the predominant pathological subtype (2). Among the oncogenic drivers of NSCLC, *KRAS* mutations are highly prevalent, occurring predominantly at codons 12 and 13 (3). Although the approval of inhibitors such as sotorasib has transformed the therapeutic landscape for the *KRAS* G12C subtype, there remains a lack of highly selective targeted agents for other common variants, including G12V, G12D, and G13C. Patients harboring these non-G12C mutations continue to rely on regimens with limited efficacy, resulting in poor prognosis. Addressing this therapeutic void is therefore a critical clinical priority. We report a case of advanced primary lung adenocarcinoma harboring concurrent *KRAS* G13C and *TP53* mutations. Following a multimodal regimen consisting of platinum-doublet chemotherapy, immunotherapy, and anti-angiogenic therapy, the patient achieved a sustained progression-free survival (PFS). Through this case report and a review of the literature, we discuss potential therapeutic strategies for this specific co-mutation profile.

## 2 Case presentation

**Figure 1:**
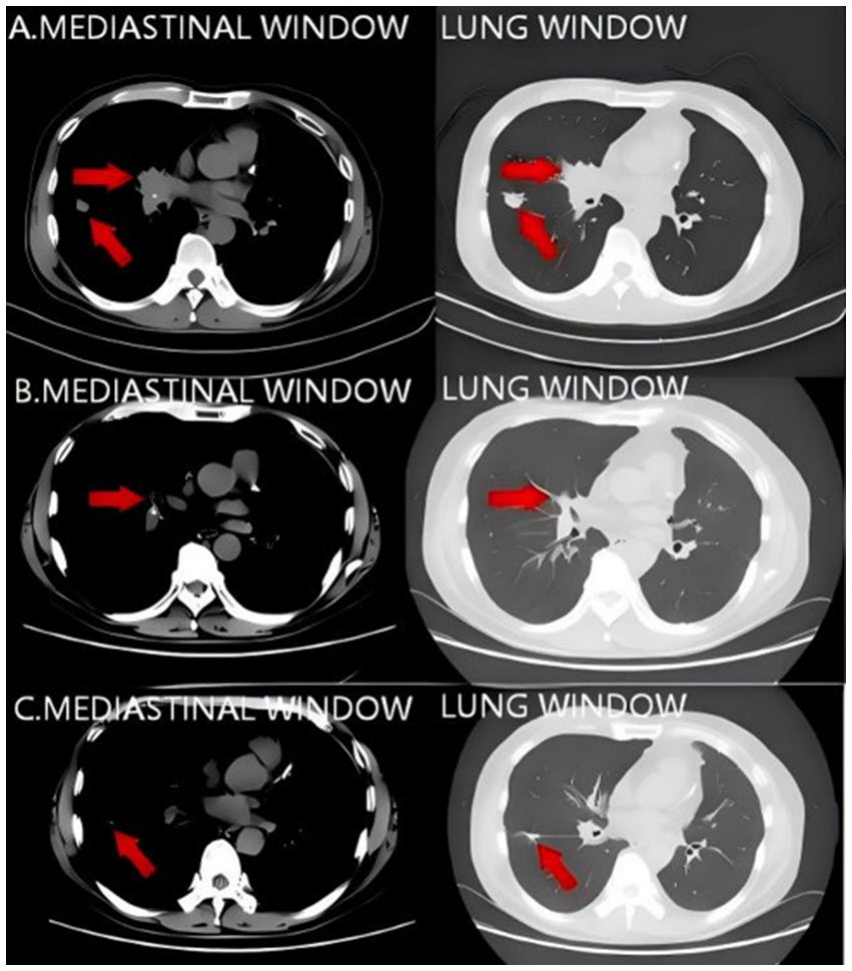
**(A)** Pulmonary CT findings on December 30, 2020. Slight enlargement of the right pulmonary hilum with consolidation of the right middle lobe, obstruction of the right middle lobe bronchial orifice, and a nodule in the right lower lobe. **(B,C)** Pulmonary CT findings on May 21, 2021. The pulmonary mass is reduced in size compared with the previous examination. Red arrows indicate the location of the tumor.

On January 4, 2021, a 59-year-old male was admitted with a 2-month history of persistent dry cough and hemoptysis. A non-contrast chest CT on December 30, 2020, revealed right hilar enlargement, right middle lobe consolidation with bronchial obstruction, and a right lower lobe nodule (Figure 1A), leading to a preliminary diagnosis of a right lung mass.

**Figure 2:**
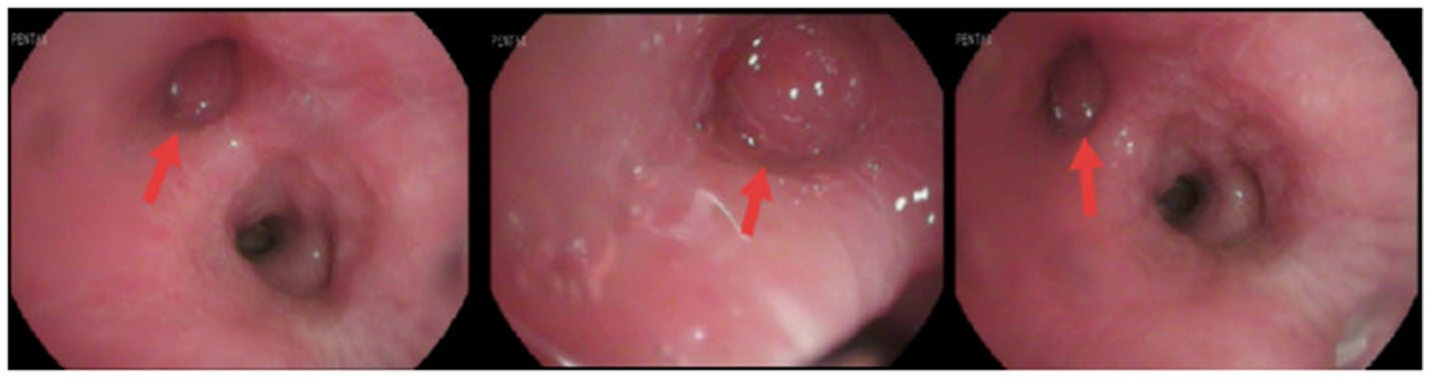
Fiberoptic bronchoscopy findings on January 6, 2021. A neoplastic lesion obstructing the orifice of the right middle lobe bronchus was observed. Red arrows indicate the location of the tumor.

**Figure 3:**
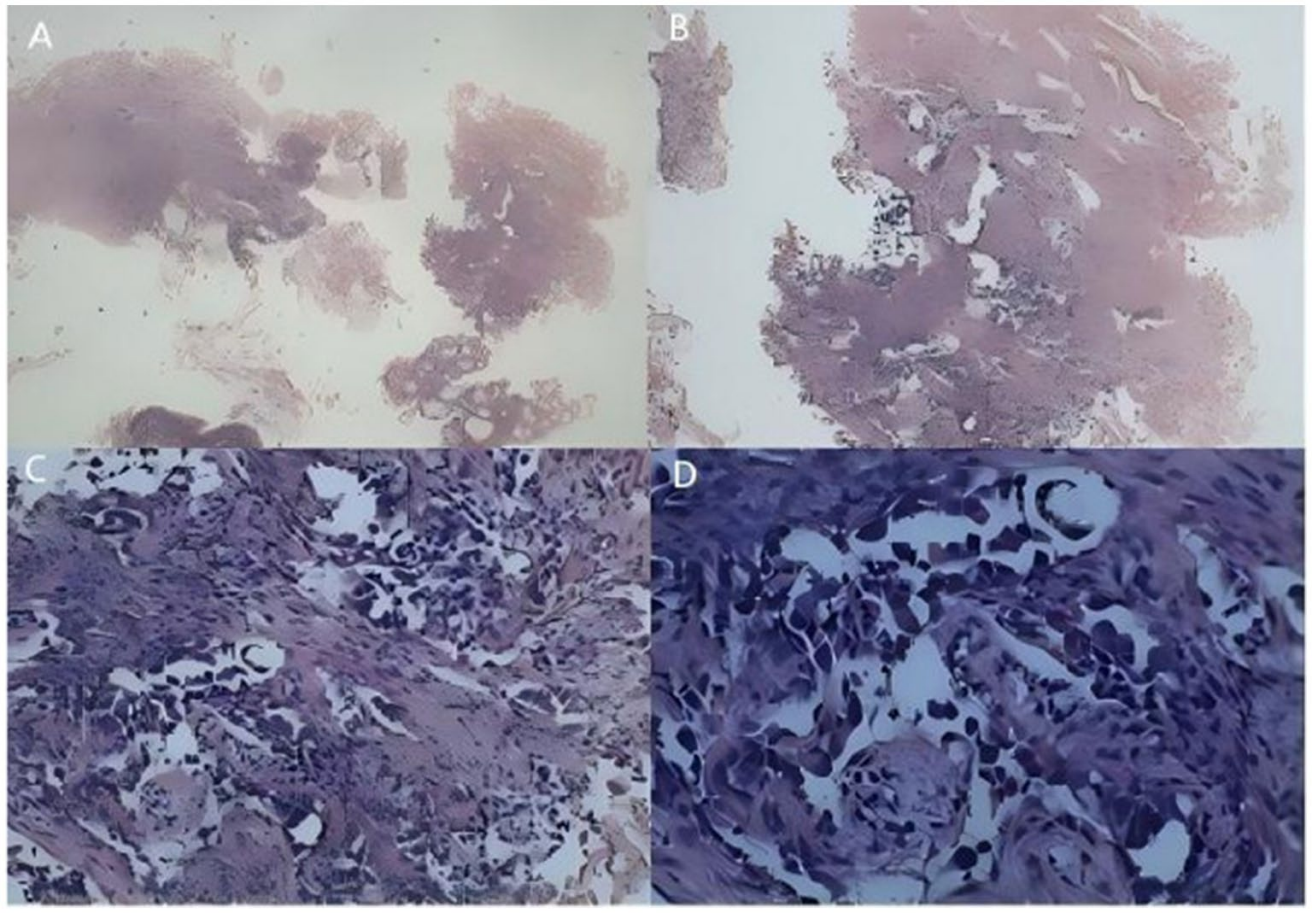
Pathological images on January 6, 2021. **(A)** Tumor tissue cells at 40× magnification. **(B)** Tumor tissue cells at 100× magnification. **(C)** Tumor tissue cells at 200× magnification. **(D)** Tumor tissue cells at 400× magnification.

The patient reported significant weight loss; review of systems was otherwise negative. Medical history was notable for pulmonary tuberculosis and former smoking (40 pack-years; quit 5 years prior), with unremarkable personal and family histories. Laboratory workup on admission demonstrated elevated high-sensitivity C-reactive protein (7.86 mg/L), microcytic hypochromic anemia (Hb 84 g/L), thrombocytosis (PLT 430 × 10^9^/L), and increased tumor markers (CEA 5.32 ng/mL; CYFRA 21-1 6.24 ng/mL). Staging scans (cranial and abdominal CT) showed no evidence of distant metastasis. Bronchoscopy identified a neoplasm obstructing the right middle lobe bronchus (Figure 2), which was biopsy-confirmed as lung adenocarcinoma (Figure 3), resulting in a diagnosis of stage IIIA NSCLC (cT4N0M0).

Although thoracic surgery was initially recommended by the multidisciplinary team, it was deemed contraindicated due to the patient’s poor pulmonary reserve. Genetic profiling identified *KRAS* p.G13C (VAF: 20.89%) and *TP53* (VAF: 12.1%) mutations. The patient underwent 6 cycles of induction therapy with carboplatin, pemetrexed, camrelizumab, and Endostar. A follow-up chest CT on May 21, 2021, demonstrated tumor regression (Figures 1B,C). Subsequently, the patient received maintenance therapy: 22 cycles of camrelizumab combined with endostar or pemetrexed (July 2021–March 2023), followed by 14 cycles of camrelizumab plus bevacizumab (March 2023–December 2024). This regimen achieved sustained disease stability for 47 months prior to treatment cessation.

**Figure 4:**
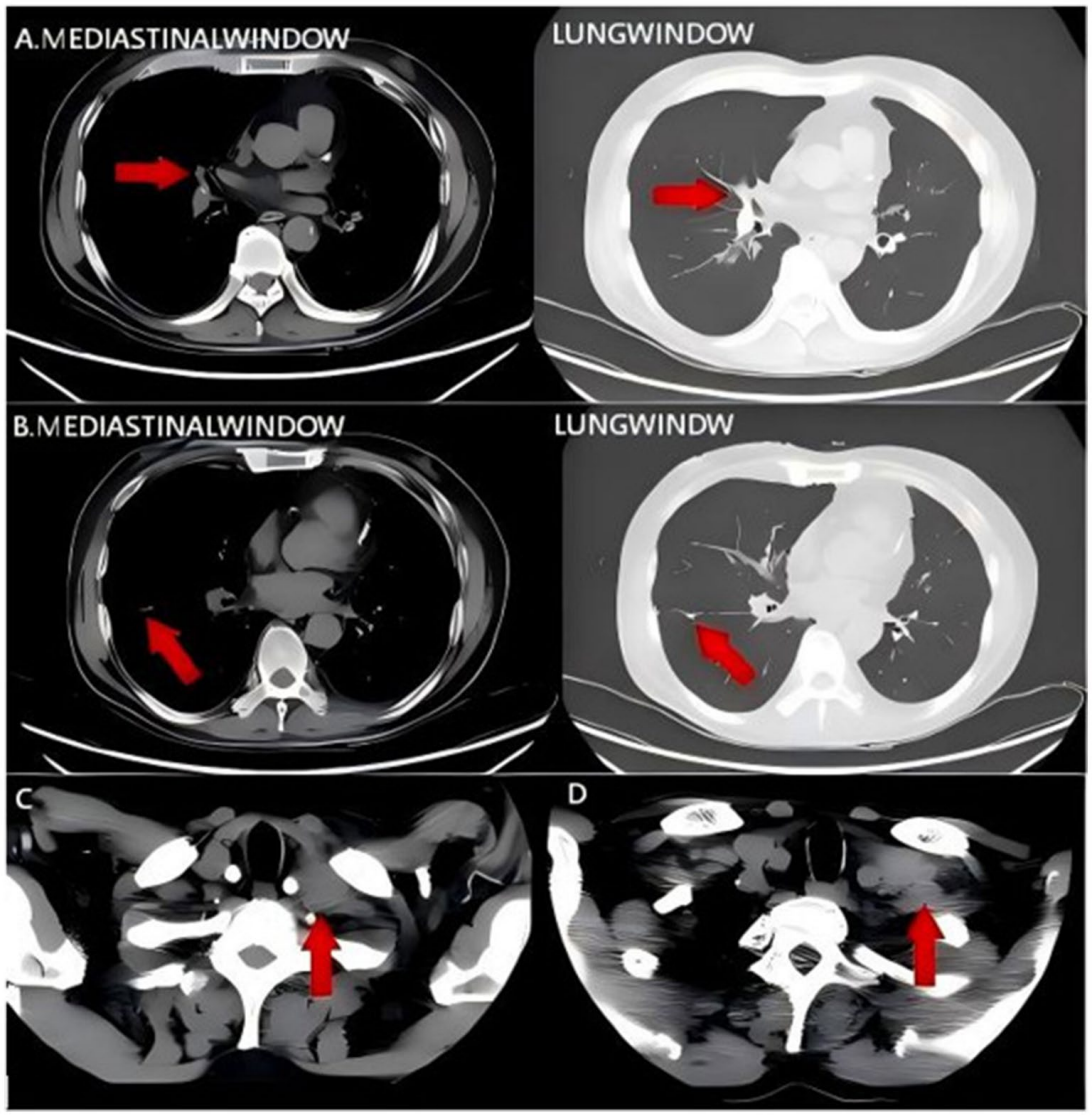
**(A–C)** Contrast-enhanced CT findings of the neck and chest on June 25, 2025. Enlarged left supraclavicular and mediastinal lymph nodes. **(D)** CT findings of the neck on August 19, 2025. Stable disease of the lymph nodes. Red arrows indicate the location of the tumor.

**Figure 5:**
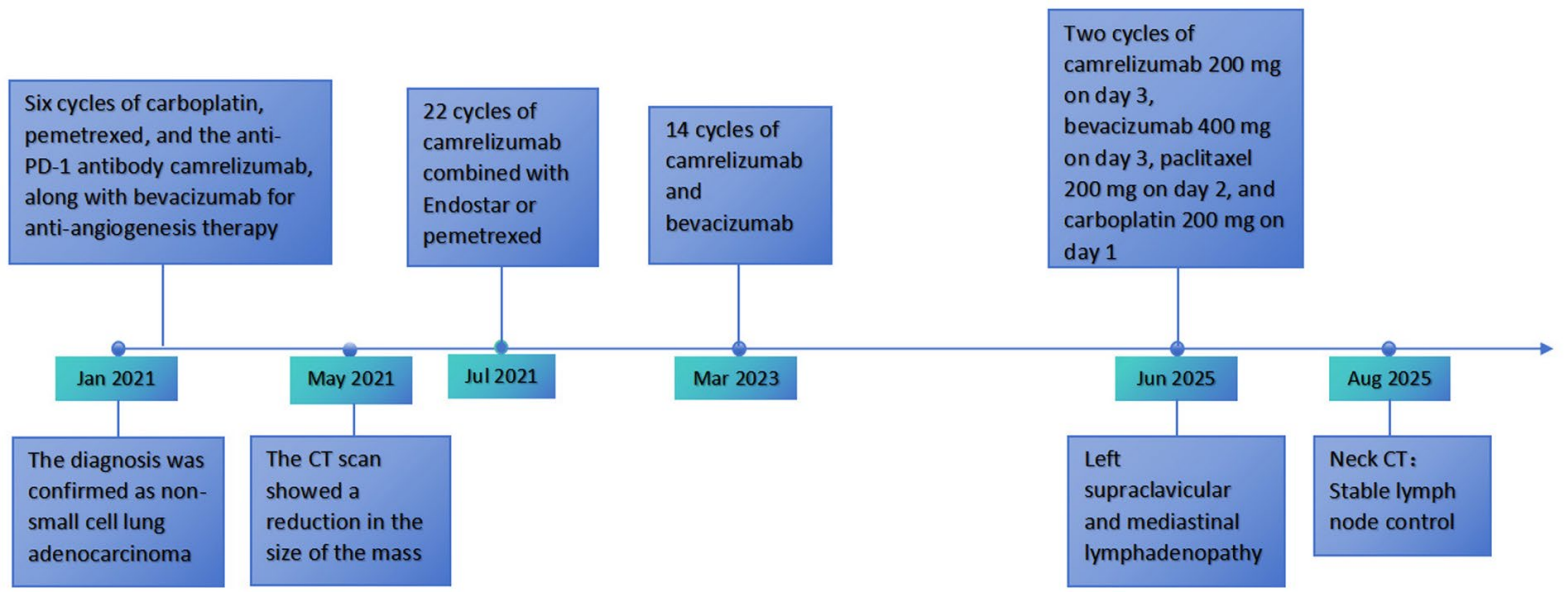
Diagnostic and treatment timeline.

In June 2025, disease progression manifested as left cervical lymphadenopathy, which was confirmed by contrast-enhanced CT (Figures 4A–C). A salvage regimen was initiated comprising carboplatin (200 mg, day 1), paclitaxel (200 mg, day 2), camrelizumab (200 mg, day 3), and bevacizumab (400 mg, day 3). By August 19, 2025, a follow-up non-contrast neck CT indicated stable disease in the involved lymph nodes (Figure 4D). The complete treatment timeline is illustrated in Figure 5. The regimen was well-tolerated; observed adverse events were mild and transient (involving hematologic, hepatic/renal, and thyroid profiles) and resolved with symptomatic management.

## 3 Discussion

We describe a rare case of NSCLC harboring concurrent *KRAS* G13C and *TP53* mutations managed with a multimodal regimen of platinum-doublet chemotherapy, immunotherapy, and anti-angiogenic agents. Despite the historically poor prognosis associated with the G13C subtype, this patient achieved an exceptional progression-free survival (PFS) of 55 months. The regimen was well-tolerated, with no grade ≥3 adverse events. To our knowledge, this represents one of the few reported instances of long-term survival in this co-mutated population utilizing this combined therapeutic strategy.

Currently, research concerning *KRAS* mutations predominantly targets the G12C variant. A comprehensive literature review of 29 clinical studies involving *KRAS* G12C-mutant NSCLC underscores the efficacy of targeted agents, specifically sotorasib and adagrasib. These therapies have yielded progression-free survival (PFS) outcomes ranging from 5.6 to 13.1 months. In terms of overall survival (OS), adagrasib demonstrated a median OS of 12.6 months, while mature OS data for sotorasib and divarasib are currently unavailable. By comparison, in the chemo-immunotherapy setting, reported PFS ranges from 4.5 to 7.2 months, with an OS range of 9.6 to 20 months (Table 1) (4–12).

**Table 1:** *KRAS* G12C-related NSCLC treatment drugs.

Drug class	Drug	*n*	Target mutation	Treatment regiment	mPFS (months)	mOS (months)
I. G12C Inhibitors	Sotorasib	345	*KRAS* G12C	Exp: Oral 960 mg once daily; Ctrl: IV 75 mg/m^2^ every 3 weeks	5.6	/
	Adagrasib	116	*KRAS* G12C	Oral adagrasib 600 mg twice daily	6.5	12.6
	Divarasib	137	*KRAS* G12C	Oral divarasib 50 mg to 400 mg once daily	13.1	/
II. Chemotherapy and combination regimens	Docetaxel	345	*KRAS* G12C	IV docetaxel 75 mg/m^2^ every 3 weeks	4.5	/
	Selumetinib	47	*KRAS* G12C or G12V	Exp: Oral selumetinib 75 mg twice daily + IV docetaxel 75 mg/m^2^ every 21 days; Ctrl: Placebo + IV docetaxel 75 mg/m^2^ every 21 days	Exp: 5.7, Ctrl: 1.4	Exp: 9.6, Ctrl: 4.4
III. Immunotherapy and combination regimens	Personalized neoantigen vaccine NEO-PV-01	38	*KRAS* G12C, G12V	NEO-PV-01 + pemetrexed, carboplatin, and pembrolizumab	7.2	Exp: 20, Ctrl: 16.8
	Immune checkpoint inhibitors (ICI)	102	*KRAS* G12C	First-line: Most received ICI; Subsequent treatments: Chemotherapy or targeted therapy	4.7	12.6
IV. New drugs	D-1553	62	*KRAS* G12C	Oral D-1553 600 mg twice daily	7.6	/
	IBI351	176	*KRAS* G12C	Oral IBI351 600 mg twice daily	9.6	/

*n*, sample size; Exp, experimental group; Ctrl, Control group; mPFS, median progression-free survival; mOS, median overall survival.

However, therapeutic options for non-G12C mutations, such as G13C, remain a clinical void. A search of the PubMed database using “*KRAS* G13C” yielded only six relevant case reports. A review of the literature (Table 2) (13–18) indicates that these cases are primarily clustered into autoimmune diseases and malignancies. Autoimmune cases are managed mainly with immunosuppression and targeted therapy, while malignancies are treated with surgery and chemotherapy. Outcomes for *KRAS* G13C-mutated autoimmune diseases are generally favorable, with PFS reaching 18 months and OS exceeding 2 years. Conversely, malignancies harboring *KRAS* G13C mutations show poor response to conventional chemotherapy, with both PFS and OS being only 2 months. Compared to previous literature, the patient in our study achieved a survival of 55 months through combined therapy, which is significantly superior to the historical average. This suggests that specific combined treatment strategies may overcome the chemotherapy resistance associated with G13C mutations.

**Table 2:** *KRAS* G13C case reports.

Disease	Sex/Age (years)	Target mutation	Symptoms	Treatment regimen	mPFS (months)	mOS (months)
I. Rosai–Dorfman disease	Male/50	G13C	Fatigue, lymphadenopathy, rash	Prednisone (PO 40 mg QD) + rituximab (intravenous 500 mg/m^2^ weekly ×4, then q2mo) Lenalidomide (15 mg QD) + dexamethasone (4 mg QD) Sirolimus (PO 4 mg titrated to 6 mg QD)	18	/
II. RAS-related LPD	Female/11	G13C	Splenomegaly, bleeding	Sirolimus (1 mg/m^2^/day PO)	/	/
III. ITP + HSP + intestinal Behçet’s	Male/3	G13C	Abd. pain, purpura, arthralgia	Prednisone (PO, dose escalated on relapse) Adalimumab (q2w × 13 mo) + prednisone taper	13	/
IV. Rosai–Dorfman + SLE	Male/15	G13C	Lymphadenopathy (SVC obstruction), pericardial effusion	Prednisone (PO) + methotrexate + azathioprine + rituximab (intravenous monthly ×6)	/	/
V. Primary sarcomatoid carcinoma of small intestine	Male/54	G13C	Abd. distension, obstruction	Surgery + chemotherapy (ifosfamide + epirubicin)	2	2
VI. RAS-related ALPS	Female/11	G13C	Fever, rash, hepatosplenomegaly	Rituximab (intravenous weekly ×4, then monthly ×11)	24	24

PO, per OS; QD, quaque die; q2mo, quaque every 2 months; q2w, quaque 2 weeks; LPD, lymphoproliferative disease; ITP, immune thrombocytopenic purpura; HSP, Henoch–Schönlein purpura; Abd, abdominal; SVC, superior vena cava; SLE, systemic lupus erythematosus; MTX, methotrexate; AZA, azathioprine; ALPS, autoimmune lymphoproliferative syndrome.

We report a patient with concurrent *KRAS* G13C and *TP53* mutations who achieved a progression-free survival (PFS) of 55 months following treatment with platinum-based doublet chemotherapy in combination with immunotherapy and anti-angiogenic therapy. In light of the clinical course observed in this case and existing evidence from the literature, the substantial clinical benefit may be attributable to biological features associated with *KRAS*/*TP53* co-mutation. As described by Skoulidis et al. (19), the “KP” subgroup represents a specific molecular phenotype characterized by a high somatic mutational burden, prominent inflammatory infiltration, and increased PD-L1 expression. Subsequent clinical studies have shown that these features translate into enhanced therapeutic sensitivity, with “KP” tumors exhibiting significantly higher objective response rates to PD-1 blockade compared with other *KRAS*-mutant subgroups (20).

Consistent with these observations, large-scale analyses by Budczies et al. (21) identified *KRAS*/*TP53* co-mutation as a stronger predictor of immune checkpoint inhibitor benefit than *KRAS*-only or wild-type tumors. Similarly, Sun et al. (22) demonstrated that *TP53* mutations are closely associated with elevated tumor mutational burden and PD-L1 expression, contributing to an inflamed tumor immune microenvironment.

In contrast, *TP53* wild-type or PD-L1-low tumors more frequently display an immunologically “cold” phenotype with limited T-cell infiltration, which may compromise the efficacy of ICI monotherapy. Importantly, however, reduced sensitivity in these settings does not imply that immunotherapy can be omitted altogether. Long-term survival observed in pivotal trials such as KEYNOTE-001 indicates that durable benefit from pembrolizumab is achievable in selected patients, underscoring that immunotherapy remains a cornerstone of treatment beyond narrowly defined biomarker-enriched subgroups (23). In *KRAS*-mutant disease, where historical outcomes with chemotherapy alone have been modest, immunotherapy represents a critical component of contemporary combination strategies.

Because the patient declined surgical intervention for personal reasons, a treatment regimen consisting of platinum-based doublet chemotherapy combined with immunotherapy and anti-angiogenic therapy was selected. Following this triplet approach, the patient achieved a clear clinical benefit, with a progression-free survival (PFS) of 55 months.

Nevertheless, as this report represents a single-case observation, the patient’s overall tumor burden was relatively lower than that typically observed in individuals with stage IV disease. While patients with stage IV disease generally present with a higher tumor burden, available evidence suggests that tumors harboring concurrent *KRAS* G13C and *TP53* mutations may exhibit a relatively favorable response to immunotherapy.

The patient described in this report belongs to the *KRAS* G13C/*TP53* co-mutated subtype. Given the relatively low incidence of *KRAS* G13C mutations in non-small cell lung cancer, large-scale clinical studies specifically addressing this subtype remain limited. Although large-scale correlation studies on *KRAS* G13C are currently unavailable, we believe that the therapeutic approach described herein could serve as a potential basis for future large-scale trials targeting this specific subtype, offering valuable reference for the clinical management of such patients. Nevertheless, the efficacy and safety profile of this strategy in a broader population require further validation through follow-up studies.

## 4 Conclusion

We report a case of *KRAS* G13C/*TP53* co-mutated NSCLC achieving prolonged survival following a combined regimen of platinum-doublet chemotherapy, immunotherapy, and anti-angiogenic therapy. These findings suggest that this triplet combination constitutes a viable therapeutic option for this specific subgroup, potentially offering durable survival benefits in clinical practice.

## Data Availability

The original contributions presented in the study are included in the article/supplementary material, further inquiries can be directed to the corresponding author.
